# Great Times for the Widows

**Published:** 1890-07

**Authors:** 


### GREAT TIMES FOR THE WIDOWS.

If things keep on very long at the rate they are going now we shall not have much left of India but the Himalayas, the Ganges, a job lot of Buddhist temples and a few rajahs with trunks full of rupees. The English have been battering away at the Hindoo superstitions ever since the days of Lord Clive, until now the Hindoo himself joins in the work of destruction. The practical representatives of John Bull had not been long in India before they objected in bluff terms to the spectacle of a lot of babies and pretty maidens sacrificed to appease the appetite of the sacred crocodile of the Ganges.

One day, a trooper raised his rifle and popped one of the crocodiles over, remarking that it was " a deuced shame to see those ugly beasts eating up all the lovely girls." So says Charles Kingsley, at least, and he is altogether too clerical to misstate the facts. It was not long before the pampered crocodile was on short rations of delicate human food, and he is probably accustomed by this time to plain boarding house fare.

The next revered institution to go was the suttee, a very reprehensible habit of India that graced the pictorial geographies twenty years ago. It was based on the wild assumption that a woman wouldn't care much to live, any how, after her liege lord died, and that the most spectacular and genteel way to interpret her wishes was to burn her alive with his body on the funeral pyre. Again the practical Britisher interposed. He must have known that unless the husbands of India were far more angelic than those of England, it was odds that a wife might take a deeper interest in life when she became a widow. At any rate, it was a deuced shame to burn a woman for no worse offense than being the relict of a Hindoo. So the suttee was wiped out.

And now comes the story that the Hindoo barbers of Bombay have completed the liberation of widows by refusing to shave their heads when the " nobler " partner dies. If a woman has a right to live, she certainly, even by Hindoo logic, has a full right to be as beautiful as she can, and the native barbers rebel at shearing her of her diadem of hair. So the Hindoos themselves have out-Englished the English in knocking their moss-grown superstitions on the head.—*N. Y. Press.*

